# The prevalence and genotypic analysis of *Toxoplasma gondii* from individuals in Scotland, 2006–2012

**DOI:** 10.1186/s13071-016-1610-6

**Published:** 2016-06-07

**Authors:** Alison Burrells, Marieke Opsteegh, Kevin G. Pollock, Claire L. Alexander, Jean Chatterton, Roger Evans, Robert Walker, Chris-Anne McKenzie, Dolores Hill, Elisabeth A. Innes, Frank Katzer

**Affiliations:** Moredun Research Institute, Pentlands Science Park, Bush Loan, Penicuik, Scotland; National Institute for Public Health and the Environment (RIVM), Centre for Infectious Disease Control, Bilthoven, The Netherlands; Health Protection Scotland, National Services Scotland, Meridian Court, Glasgow, Scotland; Scottish Parasite Diagnostic and Reference Laboratory, Glasgow Royal Infirmary, Glasgow, Scotland; Scottish Toxoplasma Reference Laboratory, Microbiology Department, Raigmore Hospital, Inverness, Scotland; Quintiles, Almondvale Way, Livingston, West Lothian Scotland; Sudden Death Brain Bank, Department of Pathology (Neuropathology) Wilkie Building Teviot Place, University of Edinburgh, Edinburgh, Scotland; USDA ARS, Northeast Area, Animal Parasitic Diseases Laboratory, Beltsville, Maryland USA

**Keywords:** *Toxoplasma gondii*, Human brains, Blood donors, Serology, Longitudinal study, Seroprevalence, Seroreversion, Genotyping

## Abstract

**Background:**

Contemporary information relating to the prevalence of *Toxoplasma gondii* in humans is lacking for the UK population, with even less information available about the human prevalence of the parasite in Scotland. To address this, two different study groups were used to determine the prevalence and genotypes of *Toxoplasma gondii* in the Scottish population.

**Methods:**

The first study group included serum samples from blood donors (*n* = 3273) over a four-year period (2006–2009) and the second study group comprised of DNA samples extracted from human brains (*n* = 151) over a five-year period (2008–2012). A *T. gondii* IgG ELISA was performed to determine seroprevalence and available sera from individuals who had seroconverted were tested by TgERP ELISA (sporozoite specific antigen). Human brain DNA was tested for *T. gondii* by ITS1 PCR and positives genotyped at the SAG3 and GRA6 loci by PCR-RFLP analysis.

**Results:**

Seroprevalence to *T. gondii* from blood donors was found to be 13.2 % (95 % CI: 11.5–15.1 %). Evidence of seroconversion (*n* = 2) as well as reversion to sero-negative status (*n* = 6) was evident from blood donors who had donated within all four collection periods (*n* = 184). The TgERP ELISA (indicating oocyst infection) was positive for one individual. The molecular detection of *T. gondii* DNA from human brains indicated a prevalence of 17.9 % (95 % CI: 12.1–24.9 %), with genotyping identifying alleles for types I and III. An increase in age was associated with an increase in detection of the parasite within both study groups.

**Conclusions:**

Our research provides current figures for the prevalence of *T. gondii* in Scotland and also shows evidence of seroreversion within the cohort of blood donors. In both study groups there was a correlation between increasing age and an increase in *T. gondii* prevalence, indicating that acquired infection plays an important role within the Scottish population.

**Electronic supplementary material:**

The online version of this article (doi:10.1186/s13071-016-1610-6) contains supplementary material, which is available to authorized users.

## Background

One third of the human population are predicted to be infected with *Toxoplasma gondii* [1]. However, current prevalence figures for the UK are limited and even less information is available specifically for Scotland, with a recent document published by the Advisory Committee on the Microbiological Safety of Food stating that there is no information about current seroprevalence rates in the UK [2]. Infection with *T. gondii* is generally asymptomatic, however those who are immunocompromised, or women who acquire the infection for the first time during pregnancy, are particularly at risk of severe disease [3]. Women who test seronegative for *T. gondii* at the beginning of pregnancy are at risk of becoming infected with the parasite during pregnancy which may result in miscarriage and future complications with the unborn child. Links have been made between *T. gondii* infection and various psychological problems, such as schizophrenia, suicide and increased risk taking behaviour [4–6]. Currently there are no firm reports in the literature linking *T. gondii* infection to these psychological conditions in humans.

The majority of seroprevalence studies have focused on pregnant women [7–9], and this specific group are not representative of the population as a whole. UK prevalence data which was published for the general population was completed up to two decades ago and studies reported a wide range of seroprevalence of between 7.7 and 40 % [2, 10]. Due to different farming practices (i.e. UK consumers are now beginning to favour meat from animals which have been raised from farms incorporating more animal friendly practices, such as outdoor reared pigs), food-producing animals may have greater exposure to environmental oocysts. Consequently, this increases their risk of becoming infected and therefore may increase the opportunity for transmission to humans from consumption of undercooked meat containing the parasite [11].

Until recently, determining whether a seropositive individual had become infected via oocysts or tissue cysts was not possible. However, recent research has shown that an ELISA incorporating the sporozoite specific antigen (*T. gondii* embryogenesis-related protein [TgERP]) can assist in identifying individuals who have been infected by ingestion of oocysts [12].

With the objective of obtaining a more up-to-date estimate for the prevalence and genotypes of human *T. gondii* infection in Scotland, the research within the current study examined two different groups of individuals: (i) serum samples from two cohorts of blood donors within Scotland over a four-year period (2006–2009); and (ii) human brain tissues from individuals who died suddenly within Scotland over a five-year period (2008–2012).

In addition to providing contemporary figures for the prevalence of *T. gondii* in the Scottish population, the samples may provide links between *T. gondii* infection and age, gender, or cause of death. The research will also identify whether seroconversion or reversion of *T. gondii* IgG has occurred, and whether recent infections were due to the tissue cysts or oocysts. Finally, there is limited information on strains that infect immunocompromised individuals (such as HIV and organ transplant patients) [13], and even less data regarding which strains infect the general population. Non archetypal and genetically distinct strains of the parasite (which have been identified in Brazil and South America), are known to be more virulent to humans (and animals) than the archetypal strains [14]. Therefore, molecular detection of the parasite from human brains will allow strain genotyping, providing information which will be more representative of the general population and will provide an indication whether any atypical strains are present.

## Methods

### Human reference sera

Reference/control sera were obtained from the Scottish Toxoplasma Reference Laboratory (Scottish Toxoplasma Reference Laboratory, Inverness, UK), who routinely test human samples for the presence of *T. gondii* IgG. The control sera supplied had previously been tested by Sabin-Feldman dye test and calibrated as international units per milliliter (IU/ml) as defined by the World Health Organisation. Control sera enabled the standardisation of an in-house *T. gondii* IgG ELISA and also acted as reference sera for testing all human sera. Controls were comprised of 125 IU/ml (strong positive), 30 IU/ml (positive), 8 IU/ml (borderline), < 2 IU/ml (negative).

### Human test sera

Serum samples were obtained from the Scottish Parasite Diagnostic Laboratory. This laboratory provided a total of 3273 serum samples from 1403 blood donors taken over four different collection periods from April 2006 until February 2009 (2006–2009) from two different cohorts (*n* = 1102 and *n* = 301). Each individual was anonymised using a specific ID number which enabled tracking of individuals throughout each collection period. Basic donor information regarding age and gender was made available from a database which had been generated by Health Protection Scotland.

### Human anti *T. gondii* IgG ELISA

In order to test the human serum samples, a human anti-*T. gondii* IgG ELISA was optimised during this study. Control sera, individual test sera and serum blank controls were tested in duplicate. This methodology was adapted from [15]. Briefly, each microwell of a Greiner Bio-One 96 well medium binding plates was coated with 100 μl of solubilized *T. gondii* RH antigen at a concentration of 6 μg/ml in carbonate buffer pH 9.6 and incubated at 4 °C overnight. Following incubation, the plate was washed 3 times with 300 μl PBS (pH7.2) containing 0.05 % Tween 20 (PBST). Control sera and test sera were diluted 1/100 in PBST and 100 μl of the dilution added to the appropriate microwell, in duplicate. Plates were incubated at room temperature (22–25 °C) for one hour. Plates were washed three times with PBST as previously, followed by addition of 100 μl conjugate (anti-human IgG alkaline phosphatase, Sigma Aldrich, Dorset, UK) to each microwell, diluted 1/1000 in PBST. Plates were incubated for one hour at room temperature. Following incubation, each well was washed 3 times in PBST as previously described and 100 μl of one-step PNPP (Thermo Scientific Pierce, Cramlington, Northumberland, UK) was added to each well. The plate was incubated in the dark at room temperature for 30 min. Optical density (OD) was measured at 405 nm using a Dynex-MRXII microplate reader (Dynex Technologies Limited, Worthing, West Sussex, UK) installed with Revelation software. OD values were corrected for blank measurements and plate to plate variation as described below.

### Corrected ELISA OD values and plate to plate variation

To analyse variation between each plate when testing serum samples using the human IgG ELISA, the methodology as described in [15] was used. Briefly, positive and negative control samples (as previously described) and serum blank controls (both tested in duplicate), were added to each plate. Once all OD readings were measured, the average OD readings for blank controls on each plate were subtracted from the OD values of the sera on that specific plate. These blank corrected OD values for the serum control samples (*n* = 8 - duplicates of each positive and negative control sample) were plotted against their appropriate average blank corrected OD value over all the plates tested (a regression line). Using the appropriate regression line the standardised blank corrected OD values for the test sera on that specific plate were calculated (their ODc value). Plates were excluded if: (i) any of the duplicates for more than one of the control samples had a coefficient of variation (CV = standard deviation of replicates/mean of replicates) above 20 %, or (ii) if the *R*^2^ value was less than 0.95. In addition, individual sera were excluded from analysis if the CV value was above 20 %, except if both duplicates of the same sample had an OD value below 0.1, as in that case, any CV value was accepted. Finally, as some of the ODc values were negative a blank value of 0.05 was added for all sera and all ODc values were ^10^log_10_-transformed (log(ODc)-values).

### Determining seroprevalence in Scottish blood donors using a binomial mixture model

In a seropositive or seronegative population, the distribution of log(ODc)-values usually follow a normal distribution [16]. This allows for analysis of the size of the seropositive and seronegative strata of the population, both of which are presumed to be log-normally distributed but with a different standard deviation and mean. A binomial mixture model takes these factors into account, and estimates two standard deviations, two means and the mixing parameter. The prevalence estimated is determined by the mixing parameter. This method does not require a cut-off to estimate population prevalence. However, when the status of individual samples is required, the mixture model can be used to determine cut-off values, as shown in the following section. The binomial mixture model was performed in Mathematica (v7.0, Wolfram Research, Illinois, USA), as described in [15]. The binomial mixture model was used to determine a cut-off value using a receiver operating characteristic (ROC) curve. This incorporated the two distributions from a binomial mixture model where the sum of sensitivity and specificity was maximized [15].

### Identification of sporozoite specific antibodies in serum samples from individuals that seroconverted

Once the cut of value had been determined, 50 μl of serum from individuals who had been identified as seronegative at one collection period, but who later became seropositive within a subsequent collection period (showing seroconversion within the previous 6–8 months), were tested for the identification of antibodies to the sporozoite specific antigen *T. gondii* embryogenesis-related protein (TgERP). This protein elicits an antibody response in humans (and also mice and pigs), who have previously been exposed to sporulated oocysts, therefore identifying individuals that have been infected with this stage of the parasite. The methodology for the ELISA is described in [12], briefly, 96 well plates were coated with 2 μg/ml TgERP in 0.1 M carbonate buffer, pH 9.6 the ELISA and interpretation of results were completed as described in [17].

### Human brain samples

Tissue samples for molecular detection of the parasite from human brains were collected (*n* = 151) over a five-year period (2008–2012) from the Medical Research Council Sudden Death Brain and Tissue Bank (MRC Sudden Death Brain and Tissue Bank, Edinburgh, Scotland - Research Ethics Committee approval, LREC 2003/8/37) over a five-year collection period (2008–2012). The Brain Bank works within the legal framework of the Human Tissue (Scotland) Act 2006 and each individual had died suddenly (no known underlying illness or disease). A total of approximately 3 g of frozen tissue was collected from three specific regions of hind brain (pons, cerebellum and medulla) for detection of parasite DNA. To access these samples a research application was submitted and approved by the South East Scotland Research Ethics Committee (NHS Lothian, Edinburgh, Scotland), reference number; REC number 11/AL/0113, IRAS project number 69852. Basic patient information was also provided with these samples to determine whether there were any specific relationships between cause of death and detection of *T. gondii*, and also to identify any trends between age and gender and parasite prevalence.

### DNA extraction, detection and genotyping of *T. gondii* from human brains

DNA was extracted from human brains using 3 g of pooled tissue (1 g of each; pons, cerebellum and medulla). Each pool was transferred into a gentleMACS M tube (Miltenyi Biotec, Woking, Surrey, UK) containing 6mls of Nuclei Lysis Solution (Promega) and samples homogenised using a GentleMacs tissue dissociator (Miltenyi Biotec). All homogenate was transferred into a 15 ml centrifuge tube and incubated overnight at 55 °C. Following incubation, samples were cooled to room temperature and 1.5 ml Protein Precipitation Solution (Promega) was added and mixed by inversion several times, then incubated on ice for five minutes. Samples were centrifuged at 1200 g for 30 min and supernatant transferred into a 15 ml centrifuge tube containing 7.5 ml isopropanol. After gentle mixing by inversion, precipitated DNA was centrifuged for ten minutes at 1000 g and the pellet washed with 2 ml 70 % ethanol, supernatant removed and pellet briefly air dried and resuspended in 700 μl distilled H_2_O. The DNA was stored at 4 °C for immediate use or at -20 °C for longer term storage.

Following the extraction of DNA from human brain tissue, samples were used to detect the presence of *T. gondii* DNA using a diagnostic nested ITS1 PCR (based on the multicopy ITS1 region [18]) and genotyping was completed on positive samples using two molecular markers (based on the single copy SAG3 and GRA6 markers [19]). The methodology for both these techniques is fully described in [20].

## Results

### Seroprevalence and determination of cut-off value using a binomial mixture model and ROC curve analysis

**Fig. 1:**
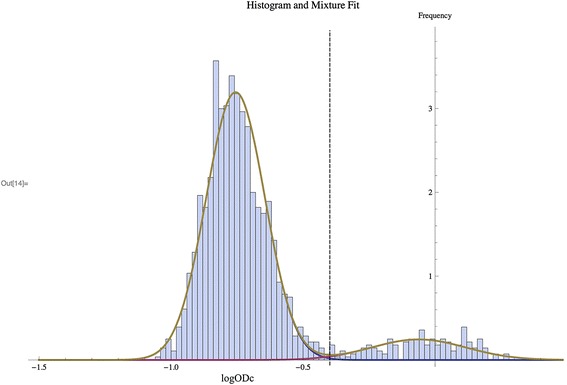
Frequency distribution of logODc values generated from Scottish blood donors (*n* = 947, period 1) using the human anti-IgG *T. gondii* ELISA. Bars represent individual blood donors, and the curved line shows the normal distributions fitted by binomial mixture analysis. The black dashed line indicates the cut-off value (log ODc -0.48) used for subsequent analysis

All results from collection period 1 (*n* = 947 individuals) were used to formulate a binomial mixture model (Fig. 1). Collection period 1 was used to produce the model, as this group contained the greatest number of individuals. Using the statistical methodology described in the methods, the binomial mixture model was fitted and distributions were plotted.

The binomial mixture model describes the overall *T. gondii* seroprevalence for the first donation period, which is estimated at 11.0 %. Performing ROC curve analysis in the data derived from the binomial mixture model results in a cut-off value of logODc = -0.48, with an estimated sensitivity of 99.0 % and specificity of 99.4 %. This cut-off value was used for further analysis of all donations; determining seropositivity with age, gender and location, across all donation periods. Using this newly defined cut-off value, *T. gondii* seroprevalence for the first donation period was estimated at 11.9 %.

### Overall seroprevalence determined by cut-off value and seroprevalence within each collection period

Using the cut-off value (logODc = -0.48), the overall prevalence of *T. gondii* during the four-year collection period (2006–2009) from participating blood donors was estimated at 13.2 % (95 % CI: 11.5–15.1 %) (185/1403) (Table 1). This figure took into account repeat donations from the same individual, including each individual only once throughout all collection periods, with a donor considered positive when a positive result was obtained for any submitted sample. This cut-off value was also used to determine seroprevalence within each collection period (Table 1) and seropositivity was similar in all collection periods. Again, repeat donations were common from blood donors throughout an individual collection period and repeat donors were counted as positive when a positive result was obtained for any submitted sample.

**Table 1:** *Toxoplasma gondii* seropositivity by collection period. Seroprevalence takes into account repeat donations from each collection period. *repeat donations taken across all collection periods where each individual is counted only once over the four-year period (2006-2009)

Period	Collection period	No. of samples (includes repeat donations) (n)	No. of individuals (no repeat donations) (n)	No. of *T. gondii* positive (no repeat donations) (n)	*T. gondii* seropositivity (no repeat donations) (mean % positive)	95 % CI (lower and upper values)
1	April 2006–July 2006	947	947	113	11.9	9.9–14.2
2	July 2006–August 2007	811	811	105	12.9	10.7–15.5
3	April 2008–September 2008	766	697	84	12.1	9.7–14.7
4	September 2008–February 2009	749	606	76	12.5	10.0–15.4
*1–4	April 2006–February 2009	3273	1403	185	13.2	11.5–15.1

### Seroprevalence of *T. gondii* from blood donors in relation to age and gender determined by cut-off value

Over the four-year period, donations were reasonably consistent throughout each age group, 30–42 age group = 28.9 % (406/1403), 17–29 age group = 28.4 %, (398/1403), 43–55 age group = 28.0 % (393/1403), 56–69 age group = 14.7 % (206/1403).

**Fig. 2:**
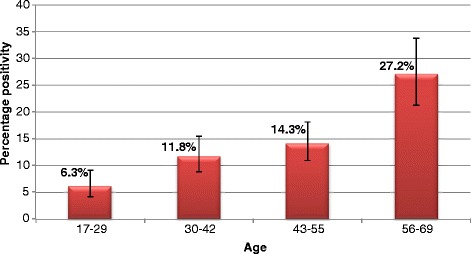
*T. gondii* seropositivity by ELISA of Scottish blood donors in relation to age (2006–2009). Error bars indicate 95 % confidence intervals

There was a clear positive relationship between increasing age and an increasing percentage of *T. gondii* seropositivity. The 56–69 age group had the highest prevalence at 27.2 % (56/206) (95 % CI: 19.5–31.7 %), whilst the lowest prevalence of 6.3 % (25/398) (95 % CI: 4.1–9.1 %) was observed in the 17–29 age group (Fig. 2).

When gender is also taken into account, donations from individuals were not biased towards either gender, with approximately an equal number of female (45.9 %, 759/1403) and male (54.1 %, 644/1403) donors. Seropositivity within males over the four-year period (2006–2009) was 12.3 % (79/644) (95 % CI: 9.8–15.1 %), compared to 13.7 % (106/759) (95 % CI: 11.6–16.3 %) in females.

### Evidence of seroconversion over a four-year period (2006–2009) and detection of sporozoite specific antibodies

Longitudinal analysis of individuals over this four-year period allowed identification of individuals and monitoring for their *T. gondii* IgG status over time (seroconversion or reversion). A total of 184 individuals donated within all four collection periods, using the cut-off value (logODc = -0.48), clear seroconversion (*T. gondii* seronegative to seropositive) was observed in 1.1 % (2/184) of individuals and reversion (seropositive to seronegative) in 3.3 % (6/184) of individuals. Not all samples were available for testing with the TgERP ELISA, however, ten individuals who had shown seroconversion within at least two subsequent blood donations were tested (Table 2).

**Table 2:** Evidence of *T. gondii* IgG seroconversion or reversion and TgERP antibody response in Scottish blood donors (2006–2009)

Donor	Positive	logODc	Collection period	Date	Status	TgERP ELISA Result (OD)
A	N	−0.50	1	May 2006		0.20
**A**	**Y**	**−0.27**	**2**	**April 2007**	**SEROCONVERTED**	**0.29**
**A**	**Y**	**−0.28**	**3**	**July 2008**		**0.19**
B	N	−0.87	1	May 2006		0.20
B	N	−0.81	2	August 2006		0.21
B	N	−1.01	4	September 2008		0.21
**B**	**Y**	**−0.02**	**4**	**January 2009**	**SEROCONVERTED**	**0.22**
C	N	−0.89	1	April 2006		0.39
**C**	**Y**	**0.25**	**3**	**May 2008**	**SEROCONVERTED**	**1.38** ^**#**^
**C**	**Y**	**0.13**	**4**	**September 2008**		**0.38**
**C**	**Y**	**0.03**	**4**	**December 2008**		**0.27**
D	N	−0.63	1	May 2006		0.22
D	N	−0.77	2	November 2006		0.17
D	N	−0.99	3	August 2008		0.12
**D**	**Y**	**0.00**	**4**	**December 2008**	**SEROCONVERTED**	**0.15**
E	N	−0.51	1	May 2006		0.17
**E**	**Y**	**0.12**	**2**	**January 2007**	**SEROCONVERTED**	**0.16**
F	N	−0.58	1	June 2006		0.18
F	N	−0.52	2	September 2006		0.22
**F**	**Y**	**−0.43**	**3**	**April 2008**	**SEROCONVERTED**	**0.20**
F	N	−0.55	3	July 2008	REVERSION	0.23
**F**	**Y**	**−0.22**	**4**	**November 2008**	**SEROCONVERTED**	**0.32**
G	N	−0.80	1	June 2006		0.15
G	N	−0.74	2	October 2006		0.24
G	N	−0.74	3	June 2008		0.28
**G**	**Y**	**−0.37**	**4**	**November 2008**	**SEROCONVERTED**	**0.23**
G	N	−0.93	4	February 2009	REVERSION	0.25
H	N	−0.66	1	July 2006		0.25
**H**	**Y**	**−0.29**	**4**	**February 2009**	**SEROCONVERTED**	**0.25**
I	N	−0.79	3	July 2008		0.14
**I**	**Y**	**−0.36**	**4**	**December 2008**	**SEROCONVERTED**	**0.14**
J	N	−0.79	3	August 2008		0.23
**J**	**Y**	**−0.08**	**4**	**December 2008**	**SEROCONVERTED**	**0.14**

Cut-off values: a) logODc cut-off value for *T. gondii* IgG ELISA = **−**0.48 b) OD cut-off value for TgERP analysis = 0.41. Key: Y = positive IgG result (and also highlighted in bold), N = negative IgG result, ^#^ = positive in TgERP ELISA

Using a positive cut-off value for the TgERP ELISA of OD 0.41, the presence of sporozoite specific antigen was identified from only one sample (donor C collection period 3, Table 2). However, two subsequent samples from this donor were TgERP negative (collection period 4, Table 2). The remaining nine donors were TgERP negative (TgERP ELISA OD < 0.41).

### Prevalence of *T. gondii* DNA in human brain tissue collected over a five-year period

Molecular detection of parasite DNA by ITS1 PCR from all samples (irrespective of age, gender or cause of death) over a five-year period (2008–2012 inclusive), has shown an overall detection rate of 17.9 % (27/151) (95 % CI: 12.1–24.0 %) (Table 3). The number of positive samples varied throughout the five-year period, with *T. gondii* DNA detected in 22.9 % (8/35) (95 % CI: 10.4–40.1 %) of individuals in 2008, 5.4 % (2/37) (95 % CI: 0.7–18.2 %) in 2009 and 15.8 % (6/38) (95 % CI: 6.0–31.3 %) in 2010. There were a greater number of positive samples in 2011 to 26.1 % (6/23) (95 % CI: 10.8–48.4 %) and in 2012 27.8 % (5/18) (95 % CI: 10.2 %–48.4 %) of samples were positive.

**Table 3:** Molecular detection of *T. gondii* DNA from human brains by year (2008–2012)

Year	No. of samples (*n*)	*T. gondii* positive (%)	95 % CI (lower and upper values)
2008	35	22.9 (*n* = 8)	10.4–40.1
2009	37	5.4 (*n* = 2)	0.6–18.2
2010	38	15.8 (*n* = 6)	6.0–31.3
2011	23	26.1 (*n* = 6)	10.2–48.4
2012	18	27.8 (*n* = 5)	9.7–53.5
2008–2012	151	17.9 (*n* = 27)	12.1–24.9

### Effect of age and gender on parasite DNA detection in human brains

Overall, a greater number of samples were obtained from males, which consisted of 79.5 % (120/151) of all individuals. This gender bias towards males was evident across all ages (data not shown). The greatest number of brain samples was obtained from the 56–69 age group, which accounted for 31.8 % (48/151; males *n* = 40, females *n* = 8) of all samples.

**Fig. 3:**
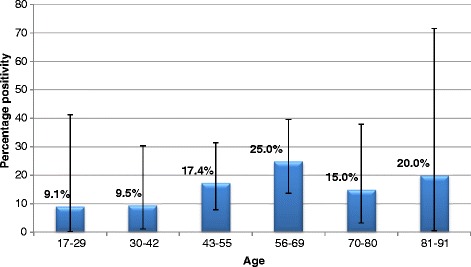
Detection of *T. gondii* DNA by ITS1 PCR in brains of humans in relation to age (2008–2012). Error bars indicate 95 % confidence intervals

Using combined results, for the five-year period, the prevalence of *T. gondii* detected in human brains increased between the ages of 17–69 (Fig. 3), however, detection levels dropped within the 70–80 age group and the 81–91 age group, however this may be due to a reduced sample size for these two age groups (*n* = 20 and *n* = 5, respectively) compared to the other age groups (17–29, *n* = 11; 30–42, *n* = 21; 43–55, *n* = 46; 56–69, *n* = 48).

### Relationship between cause of death and parasite detection

Anonymised information from the MRC Sudden Death Brain and Tissue Bank also provided details about the cause of death. The predominant cause of death for all individuals from whom samples were obtained was due to heart disease/heart attack, which accounted for 64.9 % (98/151) (95 % CI: 56.7–72.5 %) of all cases. The second most common cause of death was due to suicide (6.0 %, 9/151) (95 % CI: 1.5–8.4 %), followed by those who died due to drug overdose (5.3 %, 8/151) (95 % CI: 2.3–10.2 %).

Further analysis of cause of death and the detection of *T. gondii* DNA from the brain of each individual has highlighted that 18.4 % (18/98) (95 % CI: 11.3–27.5 %) of people who died of a heart attack were positive for *T. gondii*, which is not significantly different from positivity in people who died of other causes (9/53, 17.0 %, 95 % CI: 8.1–29.8 %, Fisher’s exact test *P* = 1.00). There were significantly fewer samples available from other cases of sudden death (Additional file 1: Table S1), therefore no comparisons between cause of death and detection of parasite DNA could be made.

### Strain genotyping using SAG3 and GRA6

**Fig. 4:**
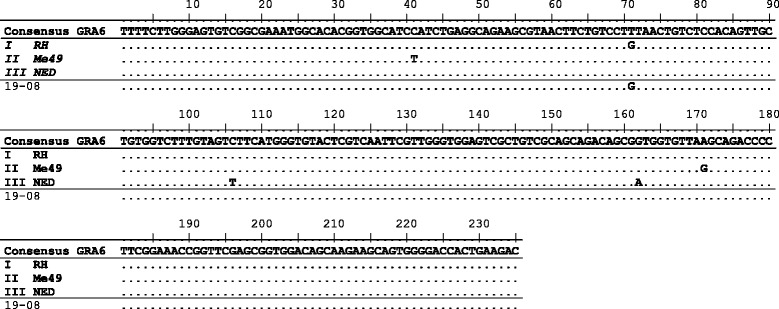
GRA6 sequence of *T. gondii* detected from a human brain. Consensus sequence indicates the nucleotides shared by a minimum of two of the three archetypal clonal lineages RH (type I), Me49 (II), and CEP (III), GenBank accession numbers AF239283, AF239285 and AF239287, respectively*.* Sequencing shows that *T. gondii* type I was identified from a human brain (sample ID 19-08), using GRA6 primers

**Fig. 5:**
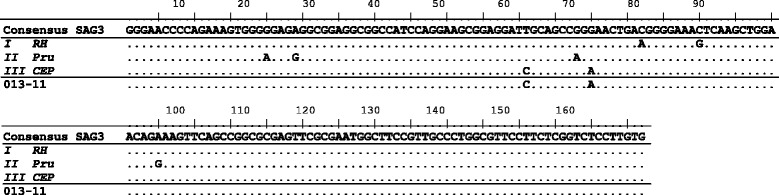
SAG3 sequence of *T. gondii* detected from a human brain. Consensus sequence indicates the nucleotides shared by a minimum of two of the three archetypal clonal lineages RH (type I), Pru (II), and CEP (III), GenBank accession numbers AF340227, AF340228 and AF340229, respectively*.* Sequencing shows that *T. gondii* type III was identified from a human brain (sample ID 13-11), using SAG3 primers

Although multiple attempts were made to amplify DNA from all individuals who were identified as positive for *T. gondii* by ITS1 PCR (*n* = 27), only three samples could be genotyped by PCR-RFLP at the SAG3 locus and one sample at the GRA6 locus (Table 4). The results identified two type III and two type I alleles from four different individuals. *T. gondii* DNA from one individual (A/08) could be sequenced at the GRA6 locus (Fig. 4), and the SAG3 locus from another individual (D/11) (Fig. 5), this confirmed the identification of *T. gondii* type I and type III alleles within the human brain tissues.

**Table 4:** *T. gondii* PCR-RFLP genotyping across two markers from four infected humans

				PCR-RFLP genotyping		
Sample ID	Gender	Age	Cause of death	SAG3	GRA6	Alleles identified
A/08	M	58	Heart attack/disease	na	I^b^	I
B/10	M	50	Heart attack/disease	III	na	III
C/10	M	27	Drug overdose	I^a^	na	I
D/11	M	58	Heart attack/disease	III^b^	na	III

^a^weak band; ^b^sequencing completed; na, no amplification

I = type I; III = type III; M = Male

## Discussion

The primary aim of this study was to obtain an estimate of *T. gondii* infection in the Scottish population using two different study groups. First, we used samples from Scottish blood donors who represent a broad age range of individuals and also includes a similar number of samples from both males and females, (as opposed to the majority of *T. gondii* seroprevalence studies which mainly focus on women of childbearing age). Secondly, prevalence was also determined by molecular detection of parasite DNA from brain tissues of individuals who had died suddenly, with no known underlying clinical disease.

When examining the seroprevalence of *T. gondii* from Scottish blood donors over a four-year period (2006–2009), the result was estimated to be 13.2 % (95 % CI: 11.5–15.1 %) (Table 1). This figure is lower but not substantially different from the prevalence determined by molecular detection in human brains over a five-year period (2008–2013), which was estimated at 17.9 % (95 % CI: 12.1–24.9 %) (Table 3), and is likely influenced by differences in study population (e.g. age distribution) and detection method. Within both study groups there appears to be a direct link between increasing positivity and increasing age (Figs. 2 and 3). Therefore, samples that have originated from older individuals is the likely reason behind the higher prevalence figure observed within human brain samples. This correlation between age and prevalence of the parasite has been described in humans and animals [21–24]. The molecular detection of the parasite from human brains initially follows this pattern (Fig. 3), however prevalence begins to fall from the 70–80 age group. The observed reduction in prevalence may be due to the reduced sample size (*n* = 20); however, without a greater sample size from this age group, it is not possible to say whether this trend would have continued.

Using blood donors allowed repeat donors to be followed longitudinally. Once the serology data was analysed using the cut-off value (log ODc = -0.48), as calculated using the binomial mixture model (Fig. 1), it was possible to identify individuals who, over time, had become seropositive or had reverted back to seronegative status. When examining individuals who had donated within all four collection periods (*n* = 184) seroreversion was greater than seroconversion (3.3 % (6/184) and 1.1 % (2/184) respectively), which is an occurrence rarely reported within the current literature. TgERP ELISA was performed on available samples that had shown seroconversion over at least two subsequent donations (*n* = 10). One positive serum sample (Table 2, donor C) was identified, indicating that this individual had been infected by ingestion of sporulated oocysts other than infection with bradyzoites (tissue cysts). It was also observed that following the positive result from the *T. gondii* IgG ELISA, the log ODc value for this sample (collection period May 2008), was greater than any other sample tested (log ODc = 0.25) as shown in Table 2. Antibodies to the sporozoite antigen could not be detected in the remaining samples which could indicate that these individuals were infected with tissue cysts. Another reason for a negative result could be because the level of TgERP antibody has dropped below a detectable level, as these antibodies are described to persist for 6–8 months [12], or that individuals were infected with such a low number of oocysts that although an IgG response against the tachyzoite stage was established, this contained too few sporozoites to generate an antibody response to the TgERP protein. In comparison to the sample which gave a positive result for the sporozoite antigen, the log ODc values following the *T. gondii* IgG ELISA using tachyzoite antigen were lower for these individuals which may have affected the sensitivity of the TgERP assay.

In conjunction with the collection of human brain tissues from the Sudden Death Brain Bank, the cause of death for each individual was recorded (in addition to age and gender). Cause of death included those who had committed suicide, had been involved in fatal road traffic accidents, but also included a broad range of other factors which can cause sudden death (e.g. heart attack, brain haemorrhage and stroke). The results showed no clear association between cause of death and molecular detection of *T. gondii*. One of the most frequent causes of death for the UK population is heart attack, second only to cancer [25] and the majority of brain samples came from cases of sudden death due to heart attack/disease [64.9 % (98/151) (95 % CI: 56.7–72.5 %)]. In addition, the percentage positivity of individuals who died of a heart attack, who also had *T. gondii* DNA detected in their brain, was 18.4 % (18/98) (95 % CI: 11.3–27.5 %), this figure is not significantly different from the overall prevalence of 17.0 % (9/53) (95 % CI: 8.1–29.8 %) in those that died of other causes of sudden death. Therefore, it is unlikely that the prevalence of *T. gondii* has any role or association with heart attack/disease. Nevertheless, several publications have reported the potential involvement with *T. gondii* and complications with the heart [26–28], and more recently a publication described a possible link to acute myocardial infarction (AMI) and *T. gondii* infection [29], where 66.7 % (32/48) of cases of AMI were also associated with the presence of anti-*T. gondii* IgG. However, any possible link between the parasite and heart complications will require further investigation, and would need to take into account age, diet and lifestyle habits.

Results from both the serological and molecular detection of the parasite provide more contemporary information on the prevalence of the parasite within the Scottish population. The results from the serology study show that there is very little variation between seroprevalence and each collection period (2006–2009), which varied from 11.9 % (113/947) (95 % CI: 9.9–14.2 %) in collection period 1 to a maximum of 12.9 % (105/811) (95 % CI: 10.7–15.5 %) in collection period 2 (Table 1). In contrast, the prevalence determined by molecular detection of the parasite varied from year to year, with 5.4 % (2/37) (95 % CI: 0.6–18.2 %) being the lowest level recorded in 2009 and 27.8 % (5/18) (95 % CI: 12.1–24.9 %) in 2012 (Table 3). The year by year variation observed by molecular detection is likely to be due to both the inhomogeneous distribution of tissue cysts in the brain and, in the case of year 2012, too small a sample size (*n* = 18) to be considered representative of that year.

When comparing these figures to previous studies, a serological study from blood donors in Scotland (from both rural and urban areas) gave an average seroprevalence of 7.7 % (*n* = 182) [10], however these results were published in 1987. Three years after these published results, a seroprevalence study from blood donors throughout the British Isles between 1990 and 1991 was conducted, which highlighted a prevalence of between 11–40 % depending on the region (as described in [2]). When using this data and taking the average figure for Scotland only, seroprevalence is calculated at 15 %, which is similar to the seroprevalence in the current study (13.2 %) (95 % CI: 11.5–15.1 %). More recently (2012), a study looking specifically at pregnant women, which included 2610 women in London, reported a seroprevalence of 17.3 % [7]. This result is similar to our reported figure for the molecular detection of the parasite (17.9 %, 95 % CI: 12.1–24.9 %). However, in this publication, when the figure was adjusted to include only those of UK origin (67.7 %), the figure dropped to 11.9 %, again, this is similar to the overall seroprevalnce in our current study (Table 1). However, the results reported within this body of work are not only from sera and tissue collected recently, but are from both genders, across a broad age range (17–91 years), and are therefore more representative of the current population than studies carried out on pregnant women or blood donors from up to nearly three decades ago. The prevalence of *T. gondii* in Scotland appears to be low when compared to other European countries, such as Germany and France, where prevalence has been reported to be as high as 59.0 % (*n* = 4854) and 54.0 % (*n* = 13,459) respectively [30, 31]. The variation between countries is likely to be due to different consumption habits of raw or undercooked meat within these countries [32]. Although a low seroprevalence rate for *T. gondii* within a population is good (as cases of ocular toxoplasmosis due to acquired infection are less likely), it also highlights a potential risk for women to become infected (for the first time) during pregnancy.

Although strain genotyping by PCR-RFLP was not particularly successful (most likely due to genotyping based on single copy markers in combination with a high concentration of host DNA compared to a low concentration of parasite DNA), two different alleles (type I and type III) from four individuals were identified (Table 4). In addition, direct sequencing of two of these amplified PCR products, using GRA6 and SAG3 markers, further confirmed the results observed by PCR-RFLP (Figs. 4 and 5). Type II is known to be the most common genotype circulating within Europe [33] however alleles for type I and III have also been identified from carnivorous wildlife in the UK [20]. Due to the invasive process required for obtaining human tissue samples, information on parasite genotypes found in humans is limited, but it is known that specific strains (in particular atypical strains) are more likely to cause a difference in the severity of disease and hence the clinical symptoms observed [34–37], similar to what has also been reported in animal species [38, 39].

## Conclusions

Overall this research provides up-to-date information about the prevalence of the parasite in humans, data which has been reported as lacking in a recent report by the Advisory Committee on the Microbiological Safety of Food [2] and also provides a novel insight into the genotypes which may be present within the UK population. The longitudinal study allowed sera from individual blood donors to be tracked over time and has shown not only evidence of seroconversion, but also seroreversion which is rarely reported within the literature.

## Abbreviations

CI, confidence interval; CV, coefficient variation; ELISA, enzyme linked immuno-sorbent assay; GRA6, dense granule antigen 6; HIV, human immunodeficiency virus; IgG, immuno-globulin G; IRAS, integrated research application system; ITS1, internal transcribed spacer 1; IU, international units; LREC, local research ethics committee; OD, optical density; ODc, corrected optical density; PBS, phosphate buffered saline; PBST, phosphate buffered saline with tween; PCR-RFLP, polymerase chain reaction restriction fragment length polymorphism; REC, research ethics committee; SAG3, surface antigen 3; *T. gondii*, *Toxoplasma gondii;* TgERP, *T. gondii* embryogenesis-related protein; UK, United Kingdom

## Additional file

Additional file 1: Table S1. Cause of death and detection of *T. gondii* DNA in brain samples. Numbers of individuals for each specific cause of death and percentage positivity for detection of *T. gondii* parasite DNA. (DOC 54 kb)
