# Supplementary material for: The prevalence and genotypic analysis of Toxoplasma gondii from individuals in Scotland, 2006–2012
Source: Parasit Vectors. 2016 Jun 7;9:324. doi: 10.1186/s13071-016-1610-6 (PMC4895884; doi:10.1186/s13071-016-1610-6)
Supplement: Additional file 1: Table S1. — Cause of death and detection of T. gondii DNA in brain samples. Numbers of individuals for each specific cause of death and percentage positivity for detection of T. gondii parasite DNA. (DOC 54 kb) [file 13071_2016_1610_MOESM1_ESM.doc]

**Additional file 1: Table S1: Cause of death and detection of *T. gondii* DNA in brain samples. Numbers of individuals for each specific cause of death and percentage positivity for detection of *T. gondii* parasite DNA.**

| **Cause of death (as described on death certificate)** | **Total individuals (n)** | ***T. gondii* positive individuals (n)** | ***T. gondii* positive (%)** | **95% CI (lower and upper values)** |
| --- | --- | --- | --- | --- |
| Heart attack | 98 | 18 | 18.4 | 11.3 - 27.5 |
| Suicide | 9 | 1 | 11.1 | 0.3 – 48.2 |
| Drug overdose | 8 | 1 | 12.5 | 0.2 – 52.7 |
| Brain haemorrhage | 6 | 2 | 33.3 | 4.3 – 77.7 |
| Unascertained death | 4 | 1 | 25 | 0.6 – 80.1 |
| Motor neurone disease | 3 | 1 | 33.3 | 0.8 – 90.6 |
| Bronchopneumonia | 2 | 1 | 50 | 1.3 – 98.7 |
| Familial amyloid disease | 1 | 1 | 100 | 5.0 – n/a |
| Abdominal aneurysm | 1 | 1 | 100 | 5.0 – n/a |
| Asthma | 1 | 0 | 0 | n/a |
| Breast cancer | 1 | 0 | 0 | n/a |
| hypertensive haematoma | 1 | 0 | 0 | n/a |
| Intracerebral haematoma | 1 | 0 | 0 | n/a |
| Insulin overdose | 1 | 0 | 0 | n/a |
| Marfans syndrome | 2 | 0 | 0 | n/a |
| Multiple injuries | 1 | 0 | 0 | n/a |
| Pancreatic carcinoma | 1 | 0 | 0 | n/a |
| Peritonitis | 1 | 0 | 0 | n/a |
| Road traffic accident | 5 | 0 | 0 | n/a |
| Small vessel disease (brain) | 1 | 0 | 0 | n/a |
| Stroke | 1 | 0 | 0 | n/a |
| Sudden adult cardiac death | 2 | 0 | 0 | n/a |

n/a = not applicable
